# High Incidence of Diseases Endemic to the Amazon Region of Brazil, 2001–2006

**DOI:** 10.3201/eid1504.081329

**Published:** 2009-04

**Authors:** Gerson Penna, Luiz Felipe Pinto, Daniel Soranz, Ruth Glatt

**Affiliations:** University of Brasília, Brasília, Brazil (G. Penna); Serra dos Órgãos Education Foundation, Rio de Janeiro, Brazil (L.F. Pinto, D. Soranz); Federal Ministry of Health, Brasília (R. Glatt)

**Keywords:** Malaria, tuberculosis, leishmaniasis, dengue, vector-borne diseases, endemic, tropical diseases, health surveillance information systems, Brazil, synopsis

## Abstract

In Brazil, reportable diseases are the responsibility of the Secretariat of Health Surveillance of the Brazilian Federal Ministry of Health. During 2001–2006, to determine incidence and hospitalization rates, we analyzed 5 diseases (malaria, leishmaniasis [cutaneous and visceral], dengue fever, leprosy, and tuberculosis) that are endemic to the Amazon region of Brazil. Data were obtained from 773 municipalities in 3 regions. Although incidence rates of malaria, leishmaniasis, tuberculosis, and leprosy are decreasing, persons in lower socioeconomic classes with insufficient formal education are affected more by these diseases and other health inequalities than are other population groups in the region.

## Abstract 2

Vector-borne and mycobacterial diseases are a major public health problem in this region.

A structured intervention to address the most prevalent diseases endemic to Brazil started when the Oswaldo Cruz Institute in Rio de Janeiro was created in 1900 and research began (*1*). Brazil has a federative political system composed of 3 levels of government: federal (union), states, and municipalities. All are considered autonomous bodies by the Federal Constitution of 1988 and none have authority over the others. Brazil has 26 states, 27 federal districts (also known as federative units), and 5,564 municipalities. Considerable demographic disparities exist among the states on the basis of their resident populations in 2007 (*2*). The 27 federative units are divided into 5 geographic regions: North, Northeast, Southeast, South, and Central-West.

The 5 geographic regions in Brazil are analytical units that are included in any epidemiologic analyses of this country. Historically, the North and Northeast regions, which include most of the Amazon River Basin, have the greatest social inequalities and the highest prevalence of disease. Furthermore, the quality of epidemiologic data is lower in the North and Northeast regions than for other regions of Brazil.

Despite advances in the Brazilian public health system (the Single Health System [SUS]) and the stated principles of universal and equitable healthcare contained in the Brazilian Constitution, many inequalities still exist with regard to access to healthcare services and to training and distribution of healthcare professionals (*3*). For example, according to Ministry of Health data, despite having the second highest number of medical schools in the world (175, second only to India) (*4*) and accepting ≈17,000 medical students each year, Brazil has a glut of physicians in the South and Southeast regions but nearly none in >1,300 municipalities (*5*).

In Brazil, health inequities among different groups are even more striking and directly associated with social and economic conditions (*6*,*7*). Reinforcement of support networks for promotion and protection of individual and collective health is Brazil’s greatest challenge, especially for states in the Amazon region of Brazil.

Demographic density in the Amazon region of Brazil is low (4.7 persons/km^2^ in 2007); many areas are nearly bereft of healthcare facilities. Paradoxically, an intense urbanization process is taking place in the region, and estimates in 2000 showed that ≈70% of the population lived in urban areas. The urbanization trend contrasts with the rural lifestyle of traditional populations (indigenous groups, river dwellers, rubber tappers, quilombolas) in the region. Quilombolas are descendants of former slaves who escaped from slave plantations that existed in Brazil until the abolition of slavery on May 13, 1888. Because of their isolated status, their primary occupations are mineral extraction or subsistence farming.

Our study involved 5 reportable (compulsory notification) diseases (malaria, leishmaniasis [cutaneous and visceral]), dengue fever, and leprosy) of the 44 reportable diseases with national coverage and 10 diseases with sentinel surveillance in Brazil. We determined the incidence and hospitalization rates of patients with these 5 diseases in states in the Amazon region of Brazil during 2001–2006.

## Amazon Region of Brazil

**Figure 1:**
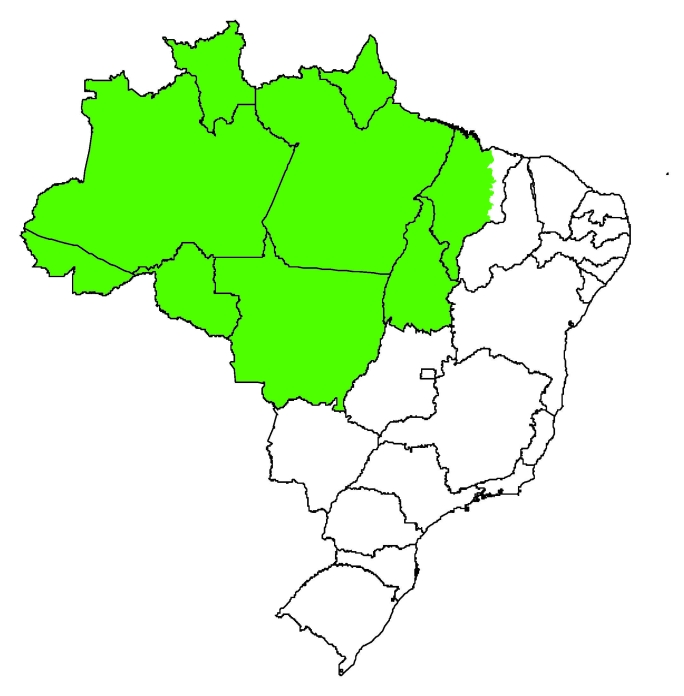
Map of Brazil showing the Amazon region (green).

The Amazon region of Brazil (Amazon River Basin), as defined by Brazilian legislation, comprises 773 municipalities in 3 geographic regions and 9 states (the entire North region, a large portion of the state of Maranhão [183 of 217 municipalities] in the Northeast region, and the entire state of Mato Grosso in the Central-West region) (Figure 1) (*8*). This region has a population of ≈23.6 million inhabitants, an area of 4.97 million km^2^ (≈60% of Brazil), and a demographic density 10× less than the national average.

The National Notifiable Disease Information System (Sistema de Informação de Agravos de Notificação Compulsória), created in 1993, is a national electronic surveillance system that contains a variety of diseases in 1 integrated database. This system accepts reports on cases and outbreaks. Relevant data are obtained from notifying health centers on standardized forms. Data are entered into the system, in most instances, by personnel from the Municipal Health Secretariats (Secretarias Municipais de Saúde). These data are transferred electronically according to a preestablished data flow: Municipal Health Secretariats → regional health coordination units (within states) → State Health Secretariat (Secretaria Estadual de Saúde) → Federal Ministry of Health.

The National Notifiable Disease Information System database, the malaria database (National Malaria Database [no longer in existence]), and Informational System of Epidemiological Surveillance of Malaria are managed and monitored by the federal government in the Secretariat of Health Surveillance/Ministry of Health (*9*). Case definitions are established by the Secretariat of Health Surveillance/Ministry of Health and are based on recommendations of the World Health Organization (*9*).

Data from the SUS Hospitalization Information System Sistema de Informações Hospitalares do Sistema Único de Saúde (SIH-SUS) were also included. These data include ≈80% of hospitalizations in the study region. This system records data according to the International Classification of Diseases, 10th revision (ICD-10) (*10*).

## Missing Data

**Figure 2:**
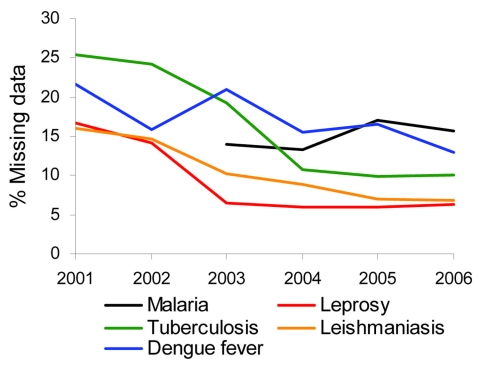
Distribution of cases of 5 diseases with missing information on patient education level among persons >10 years of age, Amazon region of Brazil, 2001–2006. Data for malaria were obtained from National Malaria Database (2003–2006); data for other diseases were obtained from the National Notifiable Disease Information System/Secretariat of Health Surveillance/Ministry of Health; population data were obtained from the Brazilian Institute of Geography and Statistics.

For the resident population >10 years of age, a detailed analysis of the proportion of cases with missing data for education level (Figure 2) showed large reductions in leishmaniasis, leprosy, and tuberculosis over the period of evaluation. However, incidence rates of 13% for dengue fever and 16% for malaria were maintained in persons for whom information on education level was not provided.

## Calculation of Indicators

Indicators (Table 1) for data analysis were calculated according to definitions of indicators and basic health data (Indicadores e Dados Básicos – Brasil – 2007; http://tabnet.datasus.gov.br/cgi/idb2007/matriz.htm) of the Inter-Agency Health Information Network, which is composed of government agencies and institutions of higher education and research (*11*). Data were analyzed by using SAS statistical software (*12*) and stratified by age group, education level, and year during 2001–2006; an aggregation of the 773 municipalities in the Amazon region of Brazil formed the basic unit of analysis. For different education levels, missing data were redistributed proportionally among all age groups >10 years of age, as per the statistical technique used.

**Table 1:** Incidence indicators for 5 reportable diseases, Brazil, 2008*

Disease	Indicator	Criteria	Method of calculation
Malaria	Annual parasitic index for malaria	No. positive examination results for malaria/100,000 residents in a given geographic area in the year under consideration (codes B50-B53; ICD-10).	(No. positive test results for malaria/total resident population) × 100,000
Leishmaniasis	Incidence rate for forms of leishmaniasis	No. new and confirmed cases of leishmaniasis (all forms)/100,000 residents in the local population of a given geographic area in the year under consideration (code B55; ICD-10)	(No. new/confirmed cases [all forms] among residents/total resident population) × 100,000
				Dengue fever	Incidence rate for dengue fever	No. new dengue fever cases (classic and dengue hemorrhagic forms)/100,000 residents in the local population of a given geographic area in the year under consideration (codes A90-A91; ICD-10)	(No. new/confirmed cases [all forms] among residents/total resident population) × 100,000
								Leprosy†	Coefficient of leprosy detection	No. newly diagnosed cases/100,000 residents in the local population of a given geographic area in the year under consideration	(No. new cases among residents/total resident population) × 100,000).
Tuberculosis	Incidence rate for tuberculosis	No. new and confirmed cases of tuberculosis (all forms)/100,000 residents in the local population of a given geographic area in the year under consideration (codes A15–A19; ICD-10)	(No. new/confirmed cases [all forms] among residents/total resident population) × 100,000

*Source: Pan American Health Organization (*11*). ICD-10, International Classification of Diseases, 10th revision. †The technical control program of the Secretariat of Health Surveillance/Ministry of Health began using a multiplication factor of 100,000 population in August 2008 to make its detection rate data for leprosy comparable with the incidence data for other diseases.

For denominators of the incidence rates, populations projected by the Brazilian Institute of Geography and Statistics during 2001–2006 were used for classification of age groups. Demographic projections were specifically developed for these population groups by education level. These projections were made by using average geometric rates of annual population growth (*13*) obtained from the 1996 population count and expanded sample data from the 2000 demographic census (*13*,*14*). We also used a correction factor for a section of persons with >12 years of formal education because the Federal Ministry of Education had indicated that during 2001–2005, the average annual increase in university enrollments in the North region, which was used as a proxy indicator for the Amazon region of Brazil, was ≈12% (*15*).

Hospitalization rates for each disease were calculated by using total registered hospital stays and the corresponding ICD-10 code as registered in the national SIH-SUS database as the numerator and the resident population as the denominator as per methods of Siqueira et al. (*16*). To facilitate comparison with other data, all indicators were adjusted to a rate/100,000 population. The total number of persons that purchased private health plans, according to data from the National Agency of Supplementary Health, was subtracted from the denominator (*17*).

## Incidence and Hospitalization Rate

Malaria was the vector-borne disease with the highest incidence in the region; the number of new cases gradually increased from 1,530/100,000 in 2001 to 2,365/100,000 in 2006. However, a reduction in the hospitalization rate for this disease was also observed and, as expected, the most affected age group was young adults 15–49 years of age (Table 2).

**Table 2:** Incidence and hospitalization rate, by age group, for 5 reportable diseases, Amazon region of Brazil*

Age group, y	2001			2002			2003			2004			2005			2006	
	Incid.	Hosp.		Incid.	Hosp.		Incid.	Hosp.		Incid.	Hosp.		Incid.	Hosp.		Incid.	Hosp.
Malaria																	
0–4	1,745.9	68.1		1,481.5	55.0		1,545.5	54.0		1,682.3	55.6		2,185.0	58.4		2,057.8	51.4
5–14	1,591.2	45.1		1,339.3	34.2		1,714.9	28.6		1,920.3	33.3		2,610.5	33.9		2,428.9	26.8
15–29†	1,464.4	87.1		1,280.5	66.9		2,227.1	63.2		2,465.4	68.6		2,967.1	70.0		2,573.0	52.9
30–49		86.5			64.9		2,118.1	59.0		2,376.7	62.3		2,869.0	60.9		2,469.6	47.0
50–69		76.7			57.1		1,558.5	48.2		1,813.2	51.8		2,326.1	57.8		2,025.7	42.7
>70		58.4			43.3		689.9	41.0		788.4	47.9		1,102.7	63.5		964.5	41.0
Total	1,529.6	72.7		1,319.4	55.5		1,898.5	50.8		2,119.2	55.0		2,661.4	56.5		2,364.9	43.8
Leishmaniasis																	
0–4	28.6	11.4		32.6	10.0		44.2	11.5		43.8	10.8		39.6	16.5		33.5	16.6
5–14	37.0	2.6		37.0	2.2		45.9	1.7		42.2	2.1		34.1	2.7		29.2	3.0
15–29	106.1	2.1		108.3	2.2		131.1	1.6		117.9	1.8		97.6	2.0		75.4	2.0
30–49	113.7	2.5		116.4	2.4		135.1	2.6		124.0	2.1		106.0	2.3		84.1	2.6
50–69	97.2	4.0		99.9	4.0		107.3	4.3		98.3	3.4		92.4	3.7		75.3	3.2
>70	68.3	58.0		66.9	43.3		76.6	40.7		68.9	47.7		62.6	63.3		61.2	41.0
Total	79.9	5.0		81.8	4.3		97.3	4.2		88.9	4.3		75.7	5.6		60.7	5.2
Dengue fever																	
0–4	86.7	8.4		48.1	13.0		57.8	15.1		29.4	10.1		38.4	13.3		44.4	12.0
5–14	152.3	21.7		96.7	30.0		112.1	32.7		52.9	21.3		87.7	28.6		85.7	25.7
15–29	347.0	55.8		199.4	72.2		235.3	74.3		123.8	50.0		197.8	65.7		165.4	51.8
30–49	423.5	64.8		259.2	85.9		298.0	81.0		165.3	55.3		256.9	72.4		221.5	56.0
50–69	358.2	82.2		251.4	109.0		275.4	109.6		154.7	71.8		227.7	91.1		216.3	68.4
>70	258.7	88.6		160.2	115.7		168.6	114.9		105.0	91.1		157.1	113.7		151.6	78.9
Total	283.8	46.6		173.0	61.6		199.6	61.8		106.6	41.8		166.4	54.6		147.9	43.3
Leprosy																	
0–4	3.2	0.0		5.6	0.0		4.6	0.1		4.3	0.0		3.3	0.0		2.7	0.1
5–14	33.1	0.6		35.5	0.7		35.1	0.6		33.5	0.5		31.4	0.2		23.5	0.4
15–29	91.4	3.6		94.1	3.9		98.0	3.5		91.1	3.6		80.8	3.4		63.0	4.7
30–49	129.8	8.6		135.3	8.7		133.3	7.5		124.7	9.0		119.0	7.2		94.8	8.0
50–69	186.7	22.4		185.6	23.4		183.6	19.8		184.8	18.8		175.6	13.0		144.9	16.1
>70	145.4	31.5		157.1	40.8		148.3	26.9		152.3	20.4		153.4	19.7		127.4	19.7
Total	84.8	5.8		87.9	6.2		88.0	5.1		83.8	5.2		78.0	4.2		61.9	5.1
Tuberculosis																	
0–4	10.8	2.3		10.8	3.1		10.2	3.8		9.4	5.5		7.9	6.4		6.9	1.1
5–14	8.7	1.7		8.4	1.3		7.6	1.6		7.4	1.5		7.4	1.4		6.5	0.6
15–29	58.0	9.0		56.2	7.6		55.5	11.6		55.8	12.8		51.5	9.4		48.2	5.8
30–49	77.7	15.8		77.1	14.8		78.2	16.3		75.5	16.8		72.7	14.5		70.0	12.5
50–69	109.3	27.6		107.9	21.9		105.3	25.4		108.6	26.8		109.7	24.0		106.4	21.2
>70	144.4	33.4		145.2	32.2		127.5	32.8		141.6	37.6		136.2	35.1		134.3	26.6
Total	51.3	10.1		50.5	8.9		49.6	10.8		49.5	11.7		47.4	10.0		45.1	7.2

*Values were standardized by the Inter-Agency Health Information Network (Pan American Health Organization, 2008) (*11*) for each disease. Malaria: annual parasitic index/100,000 population; leishmaniasis, dengue fever, and tuberculosis: incidence rate/100,000 population; leprosy: detection coefficient/100,000 population. Incid., incidence; Hosp., hospitalization rate/100,000 population. Data for malaria were obtained from the National Malaria Database (2001 and 2002) and Information System of Epidemiological Surveillance of Malaria (2003–2005), and data for the other diseases were obtained from the National Notifiable Disease Information System/Secretariat of Health Surveillance/Ministry of Health. Incidence data were obtained from the Brazilian Institute of Geography and Statistics, and hospitalization data were obtained from the Hospitalization Information System/Ministry of Health. †For malaria in 2001 and 2002, this age group corresponds to >15 years of age because the Ministry of Health used this age group to tabulate the data.

Dengue fever, which reemerged in Brazil in the 1980s, is endemic to the Amazon region of Brazil and has maintained its epidemiologic pattern of epidemics in isolated areas. In 2001, it reached its peak incidence (283.8/100,000) and became the most common vector-borne disease in the region until 2003 (Table 2).

Leishmaniasis transmission has become an urban problem, particularly in outlying areas of major and mid-sized cities in the Amazon region of Brazil. This problem is apparent despite a reduction in incidence from 79.9/100,000 in 2001 to 60.7/100,000 in 2006 and constant hospitalization rates (Table 2)

Tuberculosis incidence was lower in the study area than in the rest of Brazil (incidence rate 62/100,000 in 2006). The incidence of tuberculosis in the Amazon region of Brazil has remained constant (≈45.5 cases/100,000); concentration of cases was higher among the elderly, and the hospitalization rate was ≈10.0 (Table 2).

Leprosy showed little variation in incidence and hospitalization rates in most of the disease-endemic areas in the study region. A higher frequency was noted among adults and the elderly (Table 2).

**Figure 3:**
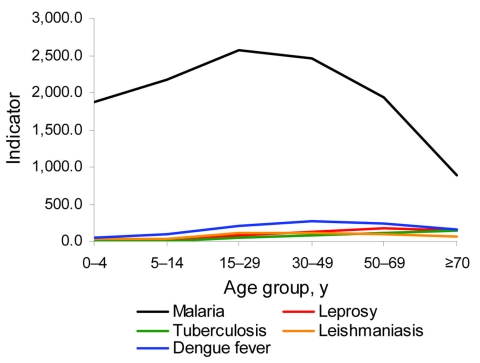
Incidence of 5 diseases by age group, Amazon region of Brazil, 2001–2006. Data for malaria were obtained from National Malaria Database (2003–2006); data for other diseases were obtained from the National Notifiable Disease Information System/Secretariat of Health Surveillance/Ministry of Health; population data were obtained from the Brazilian Institute of Geography and Statistics. Values were standardized by the Inter-Agency Health Information Network (Pan American Health Organization, 2008). Malaria: annual parasitic index/100,000 population; leishmaniasis, dengue fever, and tuberculosis: incidence rate/100,000 population; leprosy: detection coefficient/100,000 population.

We calculated disease distribution per age group. The highest incidence rates for leishmaniasis and dengue fever were among persons 30–49 years of age, the highest incidence rate for malaria was among persons 15–29 years of age, the highest incidence rate for leprosy was among persons 50–69 years of age, and the highest incidence rate for tuberculosis was among persons >70 years of age. Malaria is the most prevalent disease in the Amazon region of Brazil, although its incidence has decreased over the study period (Figure 3).

Table 3 shows incidence rates for the 5 diseases by patient education level. Resulting inequalities between various social and cultural groups are apparent. For example, the incidence of malaria is highest among groups with a lower education level.

**Table 3:** Incidence of 5 reportable diseases, by level of education, Amazon region of Brazil*

Education level, y	2001†	2002†	2003	2004	2005	2006
Malaria						
<1	–	–	3,501.2	2,141.0	2,619.9	2,578.4
1–3	–	–	3,934.6	4,199.2	5,393.4	4,420.6
4–7	–	–	1674.0	1,883.4	2,313.3	2,041.8
8–11	–	–	582.1	682.9	815.3	763.0
>12	–	–	1,405.5	1,197.3	1,502.4	1,125.9
Total	–	–	2,360.1	2,025.1	2,473.7	2,094.3
Leishmaniasis						
<1	146.0	121.3	115.9	100.0	90.7	66.6
1–3	19.9	101.0	144.8	142.6	122.3	96.0
4–7	181.0	120.0	125.1	109.1	95.2	74.2
8–11	31.2	45.1	55.3	47.8	42.0	33.9
>12	24.8	65.0	64.4	43.6	33.3	26.2
Total	95.4	95.0	108.6	95.9	80.8	62.0
Dengue fever						
<1	150.2	121.8	101.4	53.4	79.7	78.4
1–3	37.0	114.5	180.8	118.5	171.8	129.9
4–7	405.5	250.5	209.1	112.2	185.9	161.6
8–11	269.5	178.0	228.9	120.1	185.1	156.7
>12	456.8	326.1	405.0	204.2	271.4	226.9
Total	251.0	187.3	205.4	114.3	177.6	151.4
Leprosy						
<1	182.8	166.9	147.9	138.7	128.5	100.7
1–3	33.1	110.5	137.2	135.7	126.7	101.0
4–7	175.0	121.0	102.6	94.5	88.4	70.2
8–11	43.6	57.1	63.9	58.5	56.9	42.4
>12	44.8	83.2	88.5	69.7	58.9	45.4
Total	107.2	108.2	105.9	97.7	90.0	69.3
Tuberculosis						
<1	107.3	99.4	87.5	82.3	78.4	69.5
1–3	19.2	59.1	76.1	70.4	71.3	65.5
4–7	95.5	64.0	49.8	49.4	47.2	46.0
8–11	38.9	38.4	39.2	44.5	40.9	39.5
>12	43.5	71.4	55.8	54.6	49.5	42.2
Total	63.8	61.1	58.6	57.3	54.3	50.2

*Values were standardized in the Inter-Agency Health Information Network database (Pan American Health Organization, 2008) (*11*) for each disease. Malaria: annual parasitic index/100,000 population; leishmaniasis, dengue fever, and tuberculosis: incidence rate/100,000 population; leprosy: detection coefficient/100,000 population. Data for malaria were obtained from the National Malaria Database (2001 and 2002) and Information System of Epidemiological Surveillance of Malaria (2003–2005), and data for the other diseases were obtained from the National Notifiable Disease Information System/Secretariat of Health Surveillance/Ministry of Health. †This information was not systematized for malaria in the National Malaria Database in 2001 and 2002.

Leishmaniasis, even with an overall reduction in incidence in 2006, still showed differences in disease rates. The incidence rate for this disease was 3-fold greater for persons with 1–3 years of schooling than for persons with >12 years of formal education.

Dengue fever, with its predominantly urban presence, affected persons in the highest socioeconomic classes. For each year of the study, we noted an illness pattern that affected the middle and upper classes more than other socioeconomic groups.

Incidence rates for leprosy and tuberculosis were highest for groups with lowest levels of education. Incidence rates were 2× greater for groups with <1 year and 1–3 years of formal education.

## Conclusions

As is occurring in the rest of Brazil, the Amazon region of Brazil is undergoing an epidemiologic transition; infectious and parasitic diseases are decreasing and noncommunicable chronic diseases are increasing. However, vector-borne and mycobacterial diseases still constitute a public health problem in this tropical region.

Despite decreases in incidence rates of malaria, leishmaniasis, tuberculosis, and leprosy, our study shows that these diseases are more common in persons with insufficient education of lower social classes than in other population groups. This reality is more stark and systematic in the Amazon region of Brazil than in other areas of this country. According to a report of the National Commission on Social Determinants in Health (*6*), Brazil is among countries with the most skewed income distribution in the world, demonstrating the effects of social determinants as a main risk factor for illnesses in the region.

Cases of autochthonous malaria in Brazil have decreased in 2007 and 2008. Data for December 2007 showed 455,899 cases (incidence rate 1,931.40/100,000 residents). This finding represents a reduction of 2,899 hospitalizations for this disease, a decrease of 32.9% from 2006 to 2007. This decrease was caused primarily by improved control of urban malaria (*18*–*20*). Integration of disease surveillance activities into the primary care network has been the main force behind improved control of malaria.

Different forms of leishmaniasis are considered by the World Health Organization (*21*) to be a worldwide public health problem. In the Amazon region of Brazil, the incidence of leishmaniasis has decreased sharply from 108.6 cases/100,000 inhabitants in 2003 to 95.9 in 2004, 80.2 in 2005, and 62.0 in 2006; the degree of exposure was largely associated with disorganized occupation of new areas. Therefore, cases tended to occur among populations in recent land settlements and former rainforest areas. *Leishmania* (*Viannia*) *guyanensis* are protozoa enzootic to these areas. However, Campbell-Lendrum et al. (*22*) reported increasing domestication of cutaneous leishmaniasis and its possible dissemination in households in large cities in the Amazon region of Brazil.

Three serotypes of dengue virus (DEN-1, DEN-2, and DEN-3) are currently circulating in different regions of and have different regional manifestations. Brazil has the most cases of dengue fever in the Western Hemisphere; ≈70% of all cases are reported, and of this total, 17% are concentrated in the Amazon region of Brazil (*23*). When the risk factors for a dengue epidemic in Goiânia in the Central-West region of Brazil were analyzed, Siqueira et al. (*24*) noted a higher dengue prevalence among those with a lower educational level. This finding differs from our results and again highlights the specificities of disease in this region. Hospitalization rate for this disease increased for most of Brazil (*16*,*25*), but decreased in the Amazon region of Brazil during the study period from 61.6/100,000 in 2002 to 43.3 in 2006. In September 2008, data on the isolation of DEN-4 in the Amazon region were revised and now show that there is no evidence that this serotype circulates in the Amazon region of Brazil.

Two thirds of the world’s cases of tuberculosis are in Africa, the People’s Republic of China, and India (*26*). However, Brazil still has an elevated incidence rate for this disease; 42.9 cases/100,000 occurred in the Amazon region in 2007. Success in control of tuberculosis results from early detection of new cases and an effective treatment regimen. Tuberculosis incidence has remained constant in the Amazon region but the hospitalization rate for this disease has decreased. This decrease is the result of effective investment of resources in expanded access to treatment in the primary care network. This investment has been made through greater focus on family health and strategies to strengthen the local autonomy and capacity to provide quality care, together with better health surveillance and monitoring activities.

Incidence of leprosy in Brazil has shown an upward trend since 1980. However, in 2006, the number of new cases decreased to 61.9/100.000. Using negative binomial and Poisson distributions to analyze trends in the incidence of leprosy, Penna and Penna (*27*) and Martelli et al. (*28*) reported that there should have been a constant incidence, not a sharp decrease, for this disease in 2006. Leprosy is a chronic disease that is not expected to show extensive epidemiologic changes in a short period. Our results suggest that fewer reports of leprosy cases were caused by reduced new-case detection during the study period. However, hospitalization rates for this disease in 2006 are similar to those of previous years.

The SUS celebrated its 20th anniversary in 2008 and represents an ambitious example for confronting historic social inequalities in the national context through guaranteeing the right to universal healthcare. Integration of primary care and health surveillance programs has shown excellent results in the Amazon region of Brazil; particular success has occurred with malaria control, for which the number of blood slides prepared has increased from 150,000 to 450,000. Consequently, the numbers of persons who start treatment during the first 48 hours of onset of malaria symptoms has increased considerably. Conversely, factors that contribute to the presence of malaria, dengue fever, tuberculosis, leprosy, leishmaniasis, and other diseases in the Amazon region of Brazil are social inequality associated with poor funding of the public health system, imperfections in the integrated approach among the 3 spheres of government, and accelerated and disorderly urbanization of the metropolitan areas.
